# Correspondence

**Published:** 1909-05

**Authors:** 


### Correspondence.

### Castro's Method in Pleurisy.

WILMETTE, ILL., April 19, 1909.

*Dr. F. E. Daniel, Editor Texas Medical Journal, Austin, Texas.*

DEAR DOCTOR: This past winter I have successfully treated a number of cases of pleurisy by Castro's method. This dosimetric treatment has been so successful in my hands that I give it to you practically as outlined by Castro, and would be glad to have any of your readers try the method and report results.

In acute dry pleurisy, aconitine is used not as a febrifuge, but as an anti-congestant. The elements of inflammation must be attacked vigorously—congestion with aconitine and digitalin, vasomotor paralysis with strychnine—and this treatment must be continued not only until the fever has ceased, but as long as the organism will tolerate it. One granule of each may be given every fifteen minutes or at longer intervals if such is the indication.

Vesicants and blood letting are absolutely contra-indicated, the

*See *Texas State Journal of Medicine*, June, 1908, p. 39.

former because they increase the existing pain and add an inflammation to one which is already present, the latter because it weakens the patient too much and leaves him in a bad condition for undergoing the resorption of the exudates which have already been formed. Blood-letting is useful only at the time of invasion of the inflammatory state, but one is seldom called to see a patient at that time. On the other hand, leeches may be very serviceable in making the internal antiphlogistic treatment more effective. Pleuritic pains should be promptly treated with hypodermic injections of morphine, or by the application of leeches. Internally we may combine with the defervescent agents one or two granules of cicutine, as this drug depresses the motor nerve ends so as to check the impulse exciting the respiratory muscles to action and that relieves cough, fever and rapidity of the pulse.

The dose of cicutine ordinarily in these cases would be one-sixty-seventh of a grain every two or four hours, its sedative effects on the heart being watched. It must not be forgotten that pain is both a cause and an effect of inflammation. Dyspnea may follow violent pain and fever, and if these two elements are brought under control the dyspnea will cease with its causes. If special treatment for it were indicated, I should give aspidospermine. In chronic proliferate pleuritis, chronic anti-congestive means of treatment must be employed, two granules of aconitine and two of arseniate of strychnine, being used three times daily.

This form usually results from some complicating element and we may be compelled not infrequently to give two granules of the arseniate of iron and two of idoform three or four times daily as modifiers of the newly formed tissues which result from the disease. Revulsives upon the skin are also appropriate for this condition—for example: Tincture of iodine and the actual cautery, their action upon the vital forces being both general and a local one. The thoracic pains which frequently accompany this form of disease may be relieved with two granules of cicutine three or four times daily. At the end of the proliferative period we find an effusion in the form of a fibro-serous exudate. The exudate period is not of long duration, because the plura rapidly absorbs the material which is deposited upon its surface. If absorption does not go on rapidly, the exuding process being more intense than the absorptive one, or if absorption is interrupted by the formation of false membranes which are interposed between the absorbing vessels and the exuded material, the volume of the latter may become quite large.

In the former case, the pleuritis remains acute, the remedy being the same as in dry pleurisy. The fever frequently assumes an intermittent type and one of the salts of quinine is indicated. The cough, which tends to increase the inflammation, is to be treated with two granules of the hydrobromate of morphine every twenty or thirty minutes, or three granules doses of codeine given at the same intervals until the cough is allayed.

The dyspnea may result partly from fever and partly from compression. In the latter case the indication is to exert a tonic action upon the lungs, that they may be better able to accomplish their functions and resist the pressure which is caused by the effusion. Agents for accomplishing this purpose are apomorphine, hypophosphite of strychnine, and brucine, one to three granules being given every two hours.

The exudate in chronic pleuritis may be fibro-serous, or it may be purulent. If the effusion is serous, absorption may be prevented by an atonic condition of the vessels of the pleura. The circulation of the respiratory apparatus should therefore be modified with picrotoxin and the pneumogastric be incited to action by strychnine. Strychnine may be given in two or three granule doses every two hours, or apomorphine may be given in two granule doses with like quantities of caffeine, arbutin and adonidin four to six times daily for their tonic and diuretic effects.

The intermittent fever which is not uncommon in chronic pleuritis with effusion may be treated with quinine, preferably the arsenate or the salicylate. If it is necessary to perform thoracentesis, the operation should be preceded and followed by the liberal use of apomorphine and strychnine, so as to assist the pulmonary tissue in overcoming the inertia which results from prolonged compression. Pleuritis with purulent effusion is accompanied by hectic fever, anorexia, sweating, etc. Before resorting to an operation, which is about the only thing which can be done as a last resort, the activity of the digestive functions should be stimulated by the use of quassine and quinine. After the operation the arsenate of iron, quinine and strychnine should be given two or three times a day.

If the acute pleuritis is diaphragmatic, the functions of the phrenic nerve may be interfered with and a particular line of treatment required. Sedatives should be used freely with defervescents and anti-spasmodics, three granules of veratrine being given every half hour until vomiting or contro-stimulation is produced; also one granule of hyoscyamine every two hours until a decided mydriatic effect is obtained.

Now, this is about the line of treatment recommended for the different forms of pleurisy, by Castro, and while it is decidedly different from that recommended in the average text-book on medicine, I must say that I have found it extremely satisfactory and in the main I can understand why it is so. Of one thing I am very thoroughly convinced, and that is, in the first stage of pleurisy there is nothing better than this combination of aconitine, digitalin and strychnine or veratrine, and as a tonic in convalescence one can not use any remedy or combination of remedies superior to the arsenates of iron, quinine and strychnine. The more experience I have with the dosimetric method of treatment the better I like it, and I do not know but that some time I shall be a confirmed dosimetrist. At any rate, I am willing to give any method of treatment a trial and hold fast to that which is good. Wishing you great success, I am, with kind regards,

Very truly yours,

GEO. F. BUTLER.

---

### International Medical Association of Mexico.

Dr. S. H. Hodgson, President, Tampico, Tamp.

TAMPICO, TAMP., April 19, 1909.

*Dr. F. E. Daniel, "The Red Back."*

DEAR DOCTOR: Inclosed find a paper dollar to apply on my subscription account. The only objection that I have to your Journal is that it is too cheap. You furnish more reading matter for one dollar than any other journal does for twice the amount, and at the same time furnish food for reflection that lasts until the next copy comes. I hope that you will be able to attend our meeting next January in Aguascalientas and meet a bunch of about the best fellows that ever attended a medical meeting. Trusting to meet you personally during this summer, I am, fraternally yours,

S. H. HODGSON,
President.

---

CHICAGO, April 12, 1909.

*Editor Texas Medical Journal.*

MY DEAR DR. DANIEL: The following is an excerpt from an editorial appearing in the March issue of the *Kentucky Medical Journal* (A. T. McCormack, editor):

"So closely accompanying the action of the New York organization as to appear unconnected with it except to those who recognize the devious workings of the minions of the American Proprietary Association, there have appeared in the least reputable of the privately owned and nostrum supported press, simultaneous attacks on the life, character and work of our peerless National leader, George H. Simmons. That the author of these attacks [Professor Lydston] is a *specialist in the filthiest of human diseases,* seem to fit him for the degrading work assigned him, and to those who know his personal spite and venom against the great editor of the *Journal A. M. A.,* his impotence will be his excuse."

I am calling your attention to the foregoing, as it is a vile and contemptible slur upon every member of the medical profession who specializes in Urology. *"A specialist in the filthiest of human diseases";* this expression was employed to throw mud at an individual, yet it slanders *Urology,* not the *individual,* for the particular individual anonymously alluded to is exceedingly prominent and beyond any of the insinuations mentioned in the editorial. If Urology as a specialty is classed as the *"filthiest of human diseases,"* by the powers that be in the A. M. A., then the time is ripe for the Urologist to assert himself individually and collectively.

The two great bodies, the American Urological Association and the American Association of Genito-Urinary Surgeons, should consider the matter very seriously. They should seek a proper recognition of their work and should force upon our great National Association the fact that Urology is *not* a specialty in the *"filthiest of human diseases,"* but one which has accomplished as great and far-reaching results in the field of medicine and surgery as any other specialty in medicine.

I am addressing you, Doctor, for the purpose of placing this matter before every Urologist of the country, and I consider it our duty to resent such an infamous and detestable attack upon the work of the genito-urinary surgeon. Yours very truly,

LEWIS WINE BREMERMAN, M. D.

COMMENT BY THE EDITOR OF THE "RED BACK."—In presenting Dr. Bremerman's protest, it is amusing to recall the fact that this collar editor, McCormack, defends "our peerless leader," who advertised himself in the Nebraska newspapers as a "specialist in diseases of the rectum," and guaranteed a cure in every case, no matter how long standing. (See editorial page.) Are there any diseases "filthier" than diseases of the rectum? And it is worth while, too, to recall the fact that this collar editor who has such a horror of "filth" was indicted for violation of the filth ordinance of his town, and pardoned by the Governor before the trial came off. Poor devil, he has to jump as the strings are pulled, or lose his job. But it does make a difference whose ox is gored. What is sin in Lydston is virtue in Our Peerless Leader.

